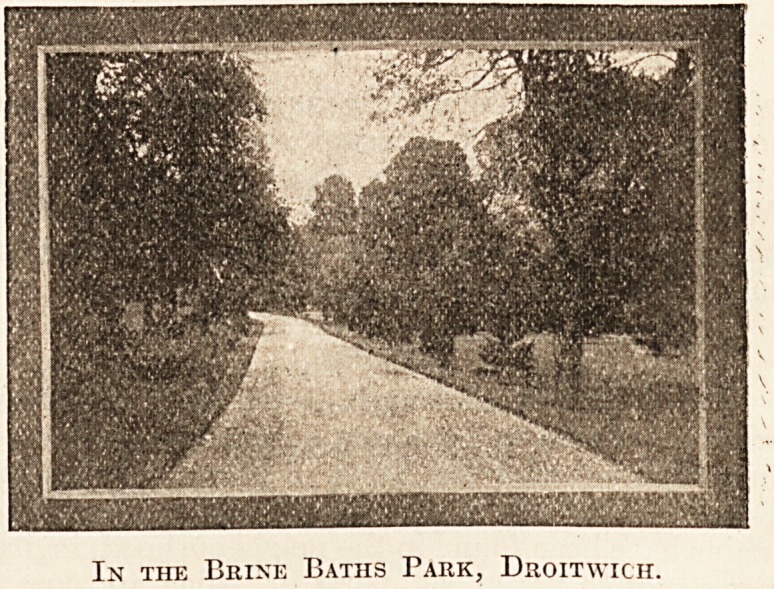# Home and Continental Spas

**Published:** 1911-02-25

**Authors:** 


### SPECIAL ARTICLES.

### HOME AND CONTINENTAL SPAS.

### II. DROITWICH.

Considered in relation to the better known spas of this country, Droitwich, with its four thousand inhabitants, is comparatively a small town; but herein lies its peculiar charm. To the invalid suffering from neurasthenia or any of the numerous nervous disorders resultant upon the "hustle" of modern life, and to the brain-worker seeking a restful and health-renewing holiday with freedom from bustle and worry, this quaint old-world town affords an ideal resort. It is 126 miles from London, six miles north-east of Worcester, and is situate amid orchards and hop-gardens, and surrounded by some of the most beautiful undulating scenery to be found in the county of Worcestershire. Being on the Great Western Railway's direct express route to Wolverhampton it is reached from Paddington in about 2½ hours, and is also easily accessible from most of the principal towns of the provinces.

The Vale of Severn, near Droitwich.

#### History.

One of the oldest boroughs in the kingdom, Droitwich is believed to have been a town of considerable importance, under the name of Salinæ, during the Roman occupation of this island. Many Roman remains have been found in the neighbourhood, including a mosaic pavement, parts of which are now to be seen in the Worcester Museum. In the Middle Ages its salt-pits were owned by the different religious houses of the district, and the Domesday Book records that King John disposed of all his property, salt furnaces, etc., in the village of Wiche, as it was then called, to the burgesses for an annual rental of £100. Whatever may be its claims to antiquity, the old narrow streets, the picturesque black and white houses, and the curious shapes of many of the cottages due to the gradual subsidence of the soil after the brine has been pumped out, make it indeed difficult to recognise in Droitwich a town of the twentieth century. The immediate neighbourhood also abounds in places of historic interest, and certainly contains some of the finest and best preserved examples of fourteenth-century architecture to be found in this country.

#### Altitude, Climate, Rainfall.

The spa is about 129 feet above sea-level and possesses a fairly bracing climate, with an absence of fogs and mists. Owing to the gentle undulations of the surrounding country and a luxuriant growth of trees, the town is well protected from the north and north-east winds, so that persons suffering from bronchial and other respiratory diseases may live here in comparative comfort, and, what is of still greater importance to visitors, the town enjoys almost complete immunity from infectious diseases. The water-supply of the spa, which is said to be one of the

Entrance of St. Andrew's Baths.

purest in the whole kingdom, is obtained direct from the Lickey Hills. The average highest temperature taken over three years is 69° 91 F., and the average lowest temperature for a similar period is 47° 08 F.

Room for Special Treatment—Douche, Needle, Aix-Douche, etc.

The average rainfall is rather less than that in most parts of the Midlands, being about 23 inches.

The Springs.

The inexhaustible supply of brine, which is pumped up from the triassic formation some 200 feet below the surface of the ground, is about twelve times the strength in saline matter of the waters of

A Cooling-room.

the ocean, and every gallon contains about 20,000 grains of solid constituents in excess of that of any other known waters. There are no other muriated springs in the whole of Europe, with the exception of Rheinfelden in Switzerland, that can be quite compared with Droitwich.

Analysis has shown the following table of constituents:—

| | |
|---|---|
| Chloride of sodium ... ... | 21761.872 |
| Chloride of magnesium ... ... | 2.560 |
| Sulphate of lime ... ... ... | 91.120 |
| Sulphate of alumina ... ... | 14.400 |
| Sulphate of soda ... ... ... | 342.720 |
| Iodide of sodium ... ... ... | .208 |
| Total salts to an imperial gallon... | 22212.880 |

The waters have been found to be active curative agents in the treatment of chronic muscular and articular rheumatism, rheumatoid arthritis, chronic, articular or irregular gout, neuritis, neuralgia, heart diseases (especially those of myocardium), neurasthenia, anæmia, chlorosis, some sclerotic diseases of the spinal cord, etc.

The Baths.

There are two bathing establishments—the Royal, opened in the year 1836, and the St. Andrew's, which is quite modern and has recently been en-

Ladies' Brine Swimming-bath.

larged and considerably improved under the able administration of Mr. J. H. Hollyer.

The accommodation provided includes reclining, needle, vapour, Aix-douche, Nauheim, Turkish and swimming baths, and other modern forms of treatment. The waiting, dressing, and cooling rooms

In the Brine Baths Park, Droitwich.

are tastefully decorated and luxuriously furnished; in fact, everything is so arranged as to render the "cure" as little irksome as possible, and to contribute to the greatest comfort of the invalid.

The high specific-gravity of the waters makes it a matter of difficulty to keep the body of the patient immersed, so that wooden bars are fitted across the ordinary baths. These baths are of teak, as the action of the brine renders any other form unsightly in a very short time. The swimming-baths afford a curious and wonderful experience; the bather floats upon the surface of the water without the slightest effort, it being practically an impossibility to sink.

The cost of treatment, of course, depends upon the kind of baths required, but the charges at Droitwich compare favourably with those at other spas. The season extends from May to October, but the baths are open and treatment can be obtained all the year round.

Accommodation.

There are several excellent hotels in close proximity to the baths, of which the Worcestershire is the largest and most palatial. There are also boarding establishments and apartment houses within a few minutes' walk to suit all classes.

Amusements and Relaxations.

The picturesque and well-wooded Brine Baths Park, immediately opposite the St. Andrew's Baths, is a great boon to visitors. Here many a pleasant hour may be spent listening to the band, which plays daily through the summer months. Plenty of comfortable seats are provided, and tennis and croquet may be played upon the broad stretches of springy turf. The bands of the Household Troops and other regiments are engaged at intervals and add much to the musical attractions. Concerts and theatrical performances by London companies are given at Salters' Hall, which also combines a pleasant reading-room and lounge. Among other amusements may be mentioned the nine-hole golf course within ten minutes' walk of the centre of the town; boating on the exceedingly pretty Droitwich Canal, and fishing in the River Salwarpe. The Worcestershire hounds are only three miles away, and the Meet often takes place close to Droitwich. In the season there are also daily excursions by char-a-banc and motor touring-cars to many historic and interesting places in the neighbourhood.

## Figures and Tables

**Figure f1:**
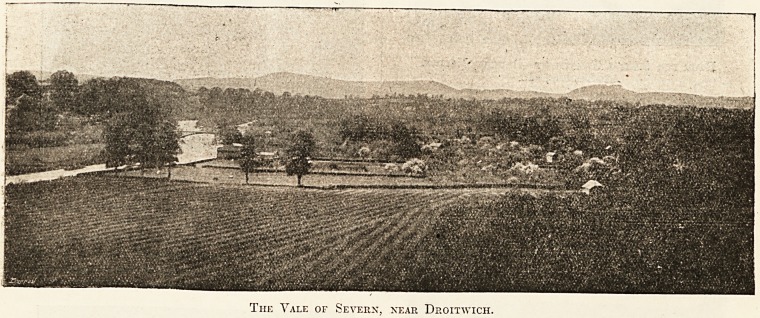


**Figure f2:**
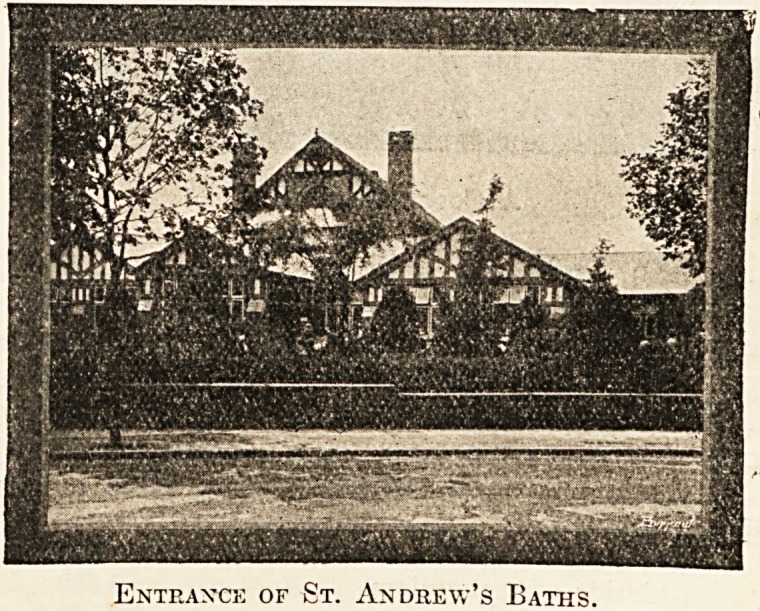


**Figure f3:**
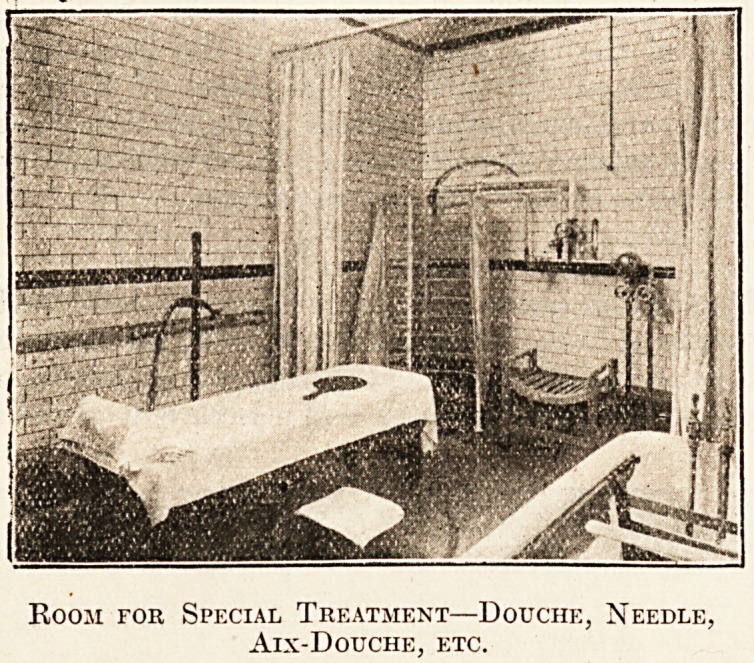


**Figure f4:**
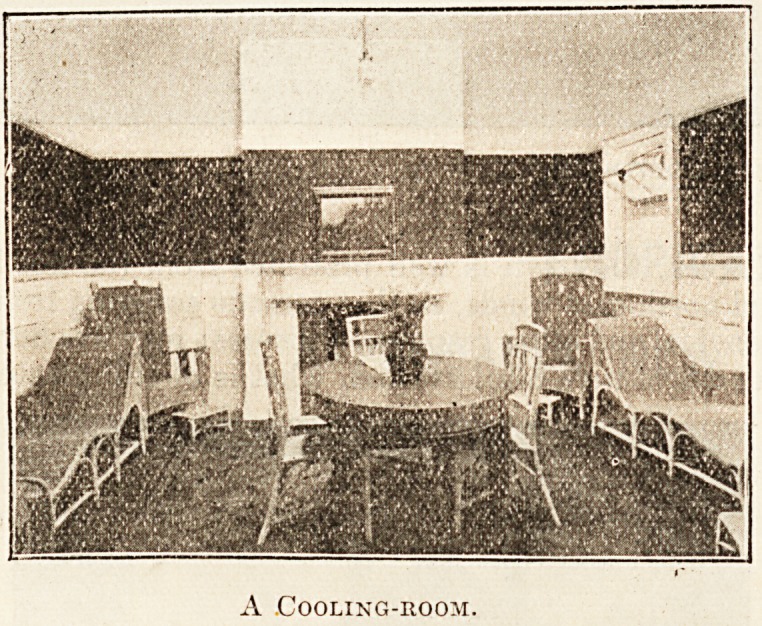


**Figure f5:**
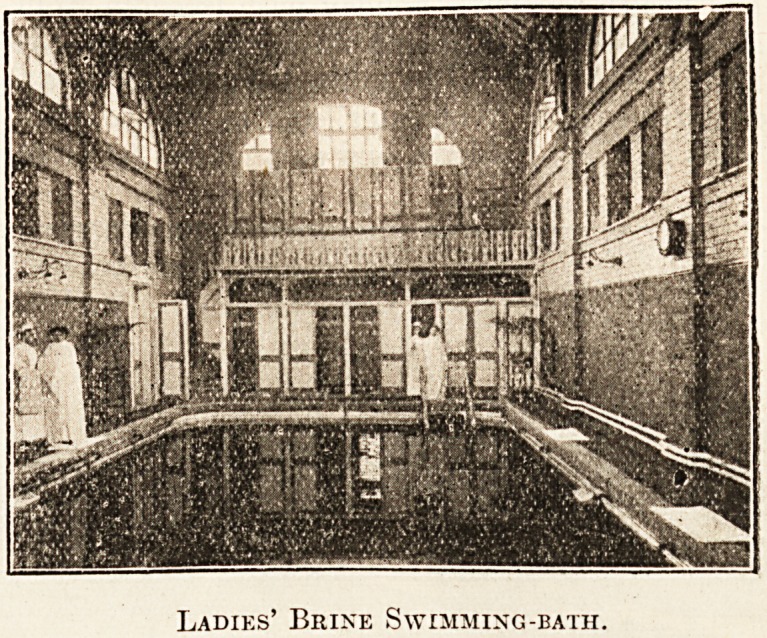


**Figure f6:**